# Integrated Functional and Histopathological Modulation of Chronic Thioacetamide-Induced Liver Fibrosis by Mesenchymal Stem Cell Therapy in a Preclinical Model

**DOI:** 10.3390/diseases14030108

**Published:** 2026-03-15

**Authors:** Anthony Brayan Rivera Prado, Luis Lloja Lozano, Daysi Zulema Diaz Obregón, Víctor Hugo Carbajal Zegarra, Joel De León Delgado, Jhon Wilfredo Pando Mayta, Alexis German Murillo Carrasco, Kelly Geraldine Yparraguirre Salcedo, Claudio Willbert Ramirez Atencio

**Affiliations:** 1 Laboratory of Regenerative Medicine, Universidad Nacional Jorge Basadre Grohmann, Avenida Miraflores S/N, Ciudad Universitaria, Tacna 23003, Peru; lllojal@unjbg.edu.pe (L.L.L.); vcarbajalz@unjbg.edu.pe (V.H.C.Z.); kyparraguirres@unjbg.edu.pe (K.G.Y.S.); 2 Innovation and Science for the Care and Support of Society—INNOVACARE, Lima 15024, Peru; daysiz.diaz.o@gmail.com; 3 Research Center for Virology, Faculty of Medicine, Universidad de San Martín de Porres, Lima 15001, Peru; jdeleond@usmp.pe; 4 Institute of Cryopreservation and Cell Therapy, Lima 15001, Peru; jpando@criocord.com.pe; 5 Immunology and Cancer Research Group (IMMUCA), Organization for Medical Innovation and Collaboration for Science OMICS, Lima 15001, Peru; agmurilloc@usp.br

**Keywords:** mesenchymal stem cells, liver fibrosis, thioacetamide, regenerative medicine, experimental animal model, histopathology, METAVIR score

## Abstract

**Background:** Chronic liver fibrosis is a progressive pathological condition characterized by excessive extracellular matrix deposition and architectural remodeling, which may ultimately lead to cirrhosis and liver failure. Although mesenchymal stem cells (MSCs) exhibit antifibrotic and immunomodulatory properties, their therapeutic effects in established chronic liver fibrosis remain incompletely defined. This study aimed to evaluate the biochemical, hematological, and histopathological effects of MSC therapy in a chronic thioacetamide-induced liver fibrosis model. **Methods:** A controlled preclinical experimental study was conducted using rats with liver fibrosis induced by intraperitoneal thioacetamide administration for 24 weeks. Animals were allocated into three groups: control, untreated fibrosis, and fibrosis treated with MSCs derived from human umbilical cord tissue after fibrosis establishment. Serum biochemical markers, hematological parameters, and liver histopathology were assessed. Fibrosis severity was evaluated using hematoxylin–eosin and Masson’s trichrome staining and graded according to the METAVIR scoring system. **Results:** Thioacetamide exposure induced chronic liver injury characterized by marked elevations in serum transaminases, reduced albumin and total protein levels, hematological alterations, and early-to-intermediate fibrosis stages (METAVIR F1–F2). MSC-treated animals exhibited approximately 40–45% reductions in transaminase levels, partial recovery of hepatic synthetic function, and attenuation of hematological alterations. Histopathological analysis demonstrated a reduction in fibrotic burden and limitation of fibrogenic progression within METAVIR F1–F2 stages. **Conclusions:** MSC therapy partially mitigates biochemical, hematological, and histopathological alterations associated with chronic thioacetamide-induced liver fibrosis, supporting its potential as a modulatory strategy to attenuate fibrogenic progression and stabilize liver function rather than as a curative intervention.

## 1. Introduction

Chronic liver fibrosis represents a major global health challenge and constitutes a common pathological pathway shared by a broad spectrum of chronic liver diseases, including viral hepatitis, metabolic-associated fatty liver disease, alcohol-related liver injury, autoimmune hepatitis, and cholestatic disorders. Persistent fibrogenic injury leads to excessive extracellular matrix deposition, progressive distortion of hepatic architecture, and deterioration of liver function, ultimately culminating in cirrhosis, portal hypertension, and hepatocellular carcinoma. These complications are associated with substantial morbidity and mortality, placing a significant burden on healthcare systems worldwide [1,2].

For many years, liver fibrosis was regarded as an irreversible process. However, growing experimental and clinical evidence has reshaped this paradigm, demonstrating that fibrosis is a dynamic and potentially reversible condition. Fibrogenesis and fibrolysis are regulated by complex and highly coordinated interactions among hepatocytes, hepatic stellate cells, immune cell populations, and components of the extracellular matrix. In early disease stages, removal of the injurious stimulus may lead to partial regression of fibrosis. Nevertheless, in advanced or long-standing chronic liver disease, spontaneous reversal is frequently incomplete, underscoring the need for therapeutic strategies capable of actively promoting tissue remodeling and restoring hepatic function [3,4].

Currently, therapeutic options for established liver fibrosis remain limited and are largely focused on controlling the underlying etiology rather than directly targeting fibrotic remodeling. Liver transplantation remains the only definitive treatment for end-stage liver disease; however, its clinical application is constrained by donor organ scarcity, perioperative risks, and the requirement for lifelong immunosuppression. In this context, regenerative medicine has emerged as a promising alternative, aiming not only to halt fibrosis progression but also to induce structural and functional recovery of the injured liver [5].

Among regenerative approaches, mesenchymal stem cells (MSCs) have gained considerable attention due to their pleiotropic biological properties, including immunomodulatory, anti-inflammatory, and antifibrotic effects. MSCs can be isolated from various sources such as bone marrow, adipose tissue, and umbilical cord, and their therapeutic potential is thought to be mediated predominantly through paracrine mechanisms rather than direct differentiation into hepatocyte-like cells. Through the secretion of bioactive factors, MSCs have been shown to modulate immune responses, inhibit hepatic stellate cell activation, reduce collagen deposition, and promote extracellular matrix remodeling, thereby contributing to fibrosis regression and tissue repair [6,7].

Despite encouraging results from preclinical and early clinical studies, important knowledge gaps persist regarding the integrated effects of MSC therapy on liver function and tissue architecture, particularly in the context of established chronic fibrosis. Many investigations have focused on isolated molecular endpoints or qualitative histological observations, often without systematically correlating structural changes with functional recovery. This lack of integrative assessment limits the interpretation of therapeutic efficacy and complicates the translation of MSC-based interventions into clinical practice.

Well-characterized preclinical models of chronic liver fibrosis are therefore essential for evaluating regenerative therapies in a controlled and reproducible manner. Chronic administration of thioacetamide has been widely employed to induce liver fibrosis, as it reliably reproduces biochemical alterations, inflammatory responses, and architectural remodeling comparable to those observed in human chronic liver disease. Importantly, this model allows for the assessment of both disease progression and therapeutic intervention, making it particularly suitable for studies exploring antifibrotic and regenerative strategies [8,9].

In this study, we aimed to comprehensively evaluate the effects of mesenchymal stem cell therapy in a chronic liver fibrosis model by integrating functional and histopathological analyses. Liver function was assessed through biochemical and hematological parameters, while tissue remodeling and fibrosis severity were evaluated using conventional histological techniques and standardized scoring systems. We hypothesized that MSC therapy induces functional hepatic improvement accompanied by histological features consistent with fibrosis regression and architectural restoration. By providing integrated functional and structural evidence, this study seeks to contribute relevant preclinical data supporting the translational potential of MSC-based regenerative therapies for chronic liver disease.

## 2. Materials and Methods

### 2.1. Study Design

This study was designed as a controlled preclinical experimental investigation aimed at evaluating the functional and histopathological effects of mesenchymal stem cell (MSC) therapy on established chronic liver fibrosis. The experimental approach integrated biochemical, hematological, and histopathological assessments to provide a comprehensive evaluation of liver injury, tissue remodeling, and regenerative response following therapeutic intervention.

A chronic liver fibrosis model was established through sustained intraperitoneal exposure to thioacetamide. Animals were randomly allocated into three predefined experimental groups with clearly defined timelines. The control group (*n* = 7) received intraperitoneal administration of physiological saline for 24 weeks and was euthanized at week 25. The fibrosis group (*n* = 7) received intraperitoneal thioacetamide for 24 weeks to induce chronic liver fibrosis and was euthanized at week 25, serving as the untreated fibrotic control. The fibrosis + MSC group (*n* = 7) received intraperitoneal thioacetamide for 24 weeks, followed by a single therapeutic administration of MSCs at week 25. Animals in this group were subsequently maintained without further intervention and euthanized at week 32 to allow evaluation of the therapeutic effects of MSCs on established liver fibrosis.

Mesenchymal stromal cells derived from human umbilical cord tissue were administered intravenously via the lateral tail vein under standard experimental conditions. This route was selected based on its widespread application in preclinical liver disease models, allowing systemic distribution and potential hepatic targeting.

Primary outcome measures included liver function parameters assessed through serum biochemical analysis and structural changes evaluated by histological and histochemical examination. Secondary outcomes included hematological parameters to assess systemic alterations associated with chronic liver injury and the regenerative effects of MSC therapy. All experimental procedures and outcome assessments were conducted according to a predefined protocol to ensure methodological consistency and reproducibility.

### 2.2. Experimental Animals

Adult female Wistar rats (*Rattus norvegicus*) were used in this study, in accordance with the approved experimental design. At the beginning of the experimental procedures, animals were approximately 12 weeks old and presented a homogeneous physiological status. Female rats were selected because chronic thioacetamide-induced liver fibrosis models have been extensively validated in female animals, demonstrating consistent fibrogenic responses and reproducible histopathological outcomes. The use of animals of similar age and physiological condition contributed to reducing inter-individual variability.

Animals were obtained from the animal facility of Jorge Basadre Grohmann National University and maintained under standardized laboratory conditions, following internationally accepted guidelines for the care and use of laboratory animals and established standards for transparent reporting in animal research [10,11]. Housing conditions included a controlled temperature of 22 ± 2 °C, relative humidity of 50–60%, and a 12 h light/dark cycle. Throughout the experimental period, animals had free access to standard laboratory chow and water ad libitum.

All procedures related to animal housing, handling, monitoring, and experimental manipulation were conducted according to standardized protocols routinely applied in preclinical research and experimental hepatology. Prior to the initiation of experimental procedures, animals underwent an acclimatization period to the housing environment. During the study, animals were monitored daily for general health status, behavior, body weight changes, and potential signs of distress throughout fibrosis induction and subsequent therapeutic intervention.

Although the estrous cycle was not synchronized, animals were randomly allocated to experimental groups and evaluated over extended experimental periods, thereby minimizing the potential influence of cycle-related variability on the overall outcomes.

Animals were randomly allocated into three experimental groups using a simple randomization approach (*n* = 7 animals per group). The sample size was defined a priori based on previous studies employing comparable chronic liver fibrosis models and regenerative therapeutic strategies and was considered sufficient to detect biologically relevant differences while complying with ethical principles of animal use. All experimental procedures were designed to comply with principles of good laboratory practice and to minimize animal suffering.

Mesenchymal stromal cells (MSCs) derived from human umbilical cord tissue were expanded under standard culture conditions and used for transplantation at passage P3. Prior to administration, cell viability was assessed using the trypan blue (Trypan Blue Solution, Sigma-Aldrich, St. Louis, MO, USA) exclusion method and consistently exceeded 90%. MSCs were administered intravenously via the tail vein at a dose of 1 × 10^6^ cells/kg body weight, suspended in phosphate-buffered saline (PBS, Gibco, Thermo Fisher Scientific, Waltham, MA, USA), pH 7.2, with a final injection volume of 0.5 mL per animal. All administrations were performed under standardized experimental conditions.

At the end of the experimental period, animals were humanely euthanized in strict accordance with internationally accepted guidelines for the care and use of laboratory animals, including the ARRIVE 2.0 guidelines and the American Veterinary Medical Association (AVMA) Guidelines on Euthanasia. Euthanasia was performed under deep general anesthesia induced by an overdose of a ketamine/xylazine mixture (ketamine hydrochloride, Zoetis, Parsippany, NJ, USA; xylazine hydrochloride, Bayer, Leverkusen, Germany), ensuring rapid loss of consciousness prior to any terminal procedure. Adequate depth of anesthesia was confirmed by the absence of corneal and pedal withdrawal reflexes, as recommended by the AVMA guidelines. Once deep anesthesia was verified, euthanasia was completed by cervical dislocation as a secondary physical method, in accordance with AVMA recommendations for small laboratory rodents, to ensure irreversible cessation of vital functions. All procedures were performed by trained and experienced personnel, and every effort was made to minimize pain, distress, and suffering throughout the process.

### 2.3. Ethical Approval

All experimental procedures involving animals constituted the first stage of a larger research project and were reviewed and approved by the Institutional Ethics Committee of the Hipólito Unanue Hospital of Tacna in November 2022. Ethical approval was granted through a Directoral Resolution (Approval Code: 841-2022-OEG-DRRHH-DR/DRS.T/GOB.REG.TACNA issued on 25 November 2022).

The experimental protocol was conducted in accordance with national regulations and internationally accepted guidelines governing the ethical use of animals in biomedical research, including the Guide for the Care and Use of Laboratory Animals [10] and the International Guiding Principles for Biomedical Research Involving Animals [12].

Animal handling and experimental procedures were designed to ensure appropriate animal welfare throughout the study. Health status was monitored regularly during fibrosis induction and therapeutic intervention, and all reasonable efforts were made to minimize animal suffering and distress, while ensuring the acquisition of scientifically valid data.

### 2.4. Induction of Chronic Liver Fibrosis

Chronic liver fibrosis was induced using thioacetamide (TAA), a hepatotoxic compound widely employed to reproduce progressive liver injury and fibrotic remodeling in experimental models. TAA (Sigma-Aldrich, St. Louis, MO, USA) was administered by intraperitoneal injection at a dose of 200 mg/kg, once per week, for a total duration of 24 weeks, following a previously established and validated protocol for chronic liver fibrosis induction in rats [9].

The TAA solution was freshly prepared prior to each administration by dissolving the compound in sterile physiological saline (0.9% NaCl, prepared in laboratory). Individual doses were adjusted weekly according to body weight to ensure consistent exposure throughout the experimental period. Control animals received intraperitoneal injections of the vehicle following the same administration schedule.

Animals were monitored regularly during the fibrosis induction phase for changes in body weight, behavior, and general health status. This chronic administration protocol resulted in progressive and persistent fibrotic changes consistent with those reported in validated TAA-induced liver fibrosis models, providing a reliable experimental platform for the subsequent evaluation of regenerative therapeutic interventions.

### 2.5. Mesenchymal Stem Cells: Source, Processing, Characterization, and Expansion

Mesenchymal stem cells (MSCs) were obtained from human umbilical cord tissue following informed consent from the donor and in accordance with established ethical and biosafety procedures. All procedures related to the isolation, processing, expansion, quality control, and immunophenotypic characterization of MSCs were performed by a specialized cell therapy facility (Instituto de Criopreservación y Terapia Celular, Criocord, Lima, Peru), following standardized protocols for therapeutic-grade cell handling.

Umbilical cord tissue was collected at birth using a sterile collection kit and transported in a dedicated transport medium containing antibiotic–antimycotic agents. The sample was delivered to the processing laboratory, where complete traceability was maintained from collection to laboratory reception. Upon receipt, the tissue was disinfected under biosafety cabinet conditions and macroscopically examined. Wharton’s jelly was isolated along the length of the cord and mechanically dissected into small fragments (approximately 2 × 2 mm). Tissue fragments were enzymatically digested using type II collagenase (2%) for 2 h at 37 °C with intermittent agitation. Enzymatic activity was neutralized using human albumin solution, and the resulting suspension was filtered, centrifuged, and resuspended in supplemented culture medium.

A representative aliquot of the processed sample was subjected to microbiological sterility testing using an automated culture system (BD BACTEC™ FX40, Becton Dickinson, Franklin Lakes, NJ, USA), confirming the absence of bacterial growth. In parallel, serological screening of the donor was performed, including HIV, hepatitis B (HBsAg and anti-HBc), hepatitis C, syphilis, HTLV I/II, and *Trypanosoma cruzi*, with all results reported as negative.

For cell expansion, the total isolated cell fraction (approximately 6.6 × 10^5^ nucleated cells) was seeded in T75 culture flasks containing supplemented Dulbecco’s Modified Eagle Medium (DMEM) and maintained at 37 °C in a humidified atmosphere with 5% CO_2_. Cell adherence, morphology, and culture conditions were monitored periodically. Medium changes were performed every 3–4 days, and cells exhibited a fibroblast-like morphology characteristic of MSCs. Upon reaching 70–100% confluence, cells were detached using TrypLE™ Express (Gibco, Thermo Fisher Scientific, Waltham, MA, USA), washed, centrifuged, and reseeded into larger culture vessels for further expansion.

Mesenchymal stromal cells (MSC) were obtained from the Cryopreservation and Cellular Therapy Institute S.A.C. (Lima, Peru), a certified cell therapy provider operating under ISO 9001 [13], ISO 13485 [14], and Good Manufacturing Practice (GMP) quality standards [15]. The MSC batch used in this study (Batch No. MSC-TAA-2023-B17; Passage P3) was immunophenotypically characterized by the supplier prior to delivery using flow cytometry (FACS Canto II, BD Biosciences, San Jose, CA, USA) in accordance with the minimal criteria established by the International Society for Cellular Therapy (ISCT) (Dominici et al., 2006) [16]. The characterization included assessment of positive MSC markers (CD73, CD90, CD105) and negative hematopoietic/immune markers (CD45, CD34, CD19, HLA-DR). The quantitative immunophenotypic profile of the MSC batch used in this study is summarized in Supplementary Table S1, and representative flow cytometry histograms are presented in Supplementary Figure S1.

### 2.6. Histological and Histochemical Analysis

At the experimental endpoint, animals were euthanized according to approved ethical procedures, and liver tissue samples were collected for histological and histochemical evaluation. Representative liver fragments were excised, gently rinsed with physiological saline to remove residual blood, and immediately fixed in buffered formalin to preserve tissue architecture.

Following fixation, liver samples were processed using standard histological techniques, including dehydration through graded ethanol series, clearing, and paraffin embedding. Paraffin blocks were sectioned at a thickness of approximately 4–5 µm using a rotary microtome, and sections were mounted on glass slides for subsequent staining.

Routine histological evaluation was performed using hematoxylin and eosin (H&E) staining, following standard histopathological procedures to assess overall liver architecture, hepatocellular morphology, inflammatory cell infiltration, sinusoidal alterations, and necrotic or degenerative changes associated with chronic liver injury [17]. Histochemical analysis was carried out using Masson’s trichrome staining to specifically visualize collagen deposition and fibrotic areas within the liver parenchyma.

The degree of liver fibrosis was assessed using an adapted METAVIR scoring system, based on the extent and distribution of fibrotic tissue observed in Masson’s trichrome–stained sections. Fibrosis staging ranged from F0 (absence of fibrosis) to F4 (cirrhosis), according to established morphological criteria [18]. The METAVIR system was applied in an adapted manner to the experimental model, focusing on histopathological features commonly used in experimental and clinical liver pathology, including fibrous septa formation, architectural distortion, and collagen deposition patterns [19].

Histological features associated with liver regeneration were evaluated qualitatively, based on morphological indicators such as restoration of lobular architecture, hepatocyte organization, and reduction of fibrotic septa. All histological and histochemical evaluations were performed using light microscopy by trained personnel blinded to the experimental groups. The assessment followed a combined qualitative and semi-quantitative approach, enabling comparative analysis of liver damage and regeneration among control, fibrotic, and MSC-treated groups.

Histological assessment of liver fibrosis was performed using established morphological criteria commonly applied in experimental and clinical liver pathology. Evaluation was based on the extent and distribution of collagen deposition, fibrous septa formation, and architectural distortion of hepatic tissue, as visualized in Masson’s trichrome– stained sections. All tissue samples were processed, stained, and analyzed under identical experimental conditions to ensure methodological consistency across experimental groups.

The degree of liver fibrosis was subsequently staged using an adapted METAVIR scoring system, applied in a blinded and standardized manner across multiple sections per animal. Fibrosis stages ranged from F0 (absence of fibrosis) to F4 (cirrhosis), according to predefined and widely validated morphological criteria. METAVIR staging was assigned exclusively based on histopathological features rather than absolute staining intensity, in line with established recommendations for fibrosis assessment.

All histological and histochemical evaluations were conducted by trained personnel blinded to experimental group allocation, in accordance with ARRIVE 2.0 recommendations for animal research to minimize interpretative bias. This combined qualitative and semi- quantitative assessment strategy ensured reproducibility and comparability of fibrosis severity between control, fibrotic, and MSC-treated groups, without reliance on digital morphometric quantification.

### 2.7. Biochemical and Hematological Analyses

At the end of the experimental period corresponding to each group, blood samples were collected for biochemical and hematological evaluation in order to assess liver function and systemic alterations associated with chronic liver injury and the therapeutic response induced by mesenchymal stem cell administration.

Blood samples were obtained at the time of euthanasia by cardiac puncture. Tubes containing EDTA were used for hematological analysis, while tubes without anticoagulant were used for serum separation for biochemical assays. Samples collected without anticoagulant were centrifuged to obtain serum, which was stored under controlled conditions until analysis.

A complete blood count was performed using an automated hematology analyzer GENRUI KT-40 (Genrui Biotech Inc., Shenzhen, China), allowing the determination of major hematological parameters, including red blood cell count, white blood cell count, hemoglobin concentration, hematocrit, platelet count, and leukocyte differential. This analysis enabled the evaluation of hematological alterations associated with chronic liver damage and systemic disease progression.

Serum biochemical analysis included the determination of parameters indicative of liver function and injury, such as aspartate aminotransferase (AST), alanine aminotransferase (ALT), alkaline phosphatase (ALP), gamma-glutamyl transferase (GGT), albumin, total proteins, and glucose. These measurements were performed using standardized enzymatic methods with a semi-automatic chemistry analyzer URIT-880 (URIT Medical Electronic Co., Shenzhen, China) and commercial reagents from QCA Química Clínica Aplicada S.A. (Amposta, Spain), following the manufacturers’ instructions.

Biochemical and hematological parameters were used as functional variables complementary to histological and histochemical findings, allowing an integrated assessment of liver injury, systemic alterations, and the therapeutic effects of mesenchymal stem cell treatment across the different experimental groups.

### 2.8. Statistical Analysis

Quantitative data are presented as mean ± standard deviation (SD). Prior to inferential analysis, data distribution was evaluated using the Shapiro–Wilk test to assess the normality of continuous variables. Given the relatively small sample size, normality testing was interpreted cautiously, and statistical analyses were conducted within an exploratory framework.

Comparisons among the three experimental groups (Control, TAA-induced fibrosis, and TAA + MSC) were performed using one-way analysis of variance (ANOVA). Statistical significance was assessed based on overall group comparisons, and a *p*-value < 0.05 was considered statistically significant.

When one-way analysis of variance (ANOVA) indicated statistically significant differences, post hoc pairwise comparisons were performed using Tukey’s multiple comparison test, which is appropriate for controlling type I error in multiple group comparisons.

Statistical analyses were conducted using R software (version 4.2.2; R Foundation for Statistical Computing, Vienna, Austria). Graphical representations were generated using the ggplot2 package. Data organization and preliminary processing were performed using Microsoft Excel (Microsoft Corporation, Redmond, WA, USA).

Results were interpreted considering both statistical outcomes and the magnitude, direction, and biological relevance of the observed changes, in coherence with biochemical, hematological, histopathological, and functional findings.

## 3. Results

### 3.1. Serum Biochemical Parameters

Serum biochemical analysis revealed marked alterations in liver function parameters following chronic thioacetamide (TAA) administration (Table 1). Animals in the TAA fibrosis group (week 25) exhibited a pronounced elevation of serum transaminases, with AST and ALT levels increased by approximately threefold and fourfold, respectively, compared to the control group, indicating severe hepatocellular injury. In parallel, cholestatic markers were markedly affected, as evidenced by a more than twofold increase in alkaline phosphatase (ALP) and a nearly fivefold elevation in gamma-glutamyl transferase (GGT), consistent with bile duct involvement and chronic hepatic damage.

**Figure 1:**
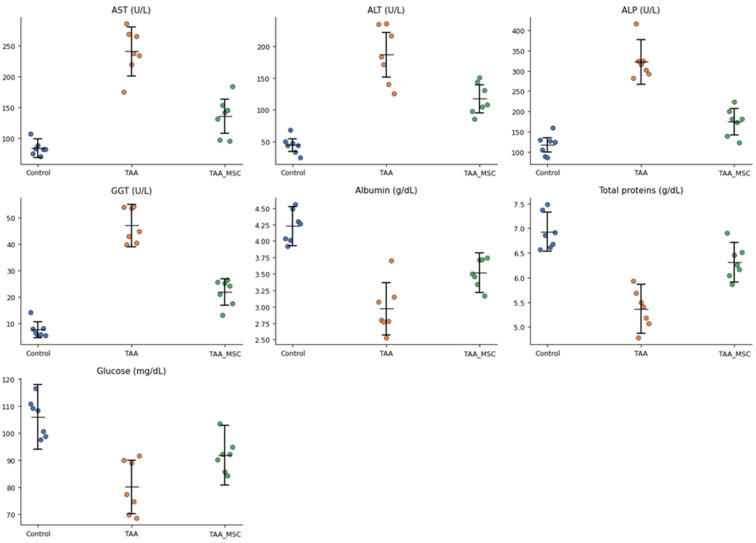
Serum biochemical parameters reflecting liver function in experimental groups. Individual serum values of aspartate aminotransferase (AST), alanine aminotransferase (ALT), alkaline phosphatase (ALP), gamma-glutamyl transferase (GGT), albumin, total proteins, and glucose are shown for control rats receiving saline (week 25), rats with thioacetamide-induced liver fibrosis (TAA, week 25), and rats treated with mesenchymal stem cells following TAA exposure (TAA + MSC, week 32). Each dot represents an individual animal (n = 7 per group). Horizontal lines indicate group means, and error bars represent ± standard deviation.

In contrast, animals receiving mesenchymal stem cell (MSC) therapy after TAA exposure (week 32) showed a partial attenuation of these biochemical alterations. AST and ALT levels were reduced by approximately 40–45% relative to the untreated TAA group, while ALP and GGT levels decreased by approximately 35–45%. However, none of these parameters returned to baseline control values, indicating an incomplete but biologically relevant improvement in liver function. These biochemical alterations are illustrated in Figure 1, which shows the distribution of individual serum parameters across the experimental groups.

Regarding hepatic synthetic function, serum albumin and total protein concentrations were reduced by approximately 30–35% in the TAA fibrosis group compared to controls, reflecting impaired protein synthesis associated with chronic liver injury. MSC-treated animals exhibited a partial restoration of albumin and total protein levels, reaching approximately 85–90% of control values. Serum glucose levels showed a moderate decrease (approximately 15–20%) in TAA-treated animals, whereas MSC therapy was associated with a tendency toward normalization.

### 3.2. Hematological Parameters

**Figure 2:**
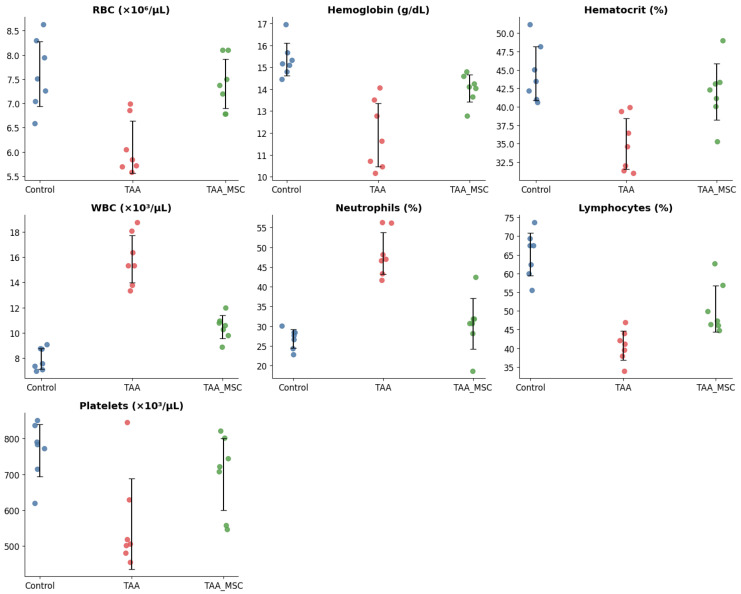
Hematological parameters across experimental groups. Individual data points represent values obtained from each animal in the Control (saline), TAA-induced fibrosis, and TAA + MSC treatment groups. Horizontal lines indicate the mean, and vertical bars represent the standard deviation (SD). Parameters evaluated include red blood cells (RBC), hemoglobin, hematocrit, white blood cells (WBC), neutrophils, lymphocytes, and platelets. TAA administration induced marked hematological alterations consistent with systemic inflammation and hepatic injury, whereas MSC treatment partially restored these parameters toward control values.

Hematological analysis revealed significant systemic alterations associated with chronic thioacetamide (TAA)-induced liver injury (Table 2; Figure 2). Animals in the TAA fibrosis group (week 25) exhibited a marked reduction in red blood cell count, hemoglobin concentration, and hematocrit values compared to control animals, consistent with the development of anemia in the context of chronic hepatic dysfunction. Red blood cell counts decreased by approximately 20–25%, accompanied by a similar reduction in hemoglobin and hematocrit levels.

In parallel, total white blood cell counts were markedly elevated in TAA-treated animals, increasing by approximately 75–80% relative to controls, indicative of a systemic inflammatory response. This leukocytosis was primarily driven by a significant increase in neutrophil percentages, which rose from approximately 28% in control animals to nearly 46% in the TAA fibrosis group, while lymphocyte proportions decreased correspondingly.

Animals receiving mesenchymal stem cell (MSC) therapy after TAA exposure (week 32) showed a partial normalization of hematological parameters. Red blood cell count, hemoglobin, and hematocrit values increased relative to the untreated TAA group, although they did not fully reach control levels. Similarly, white blood cell counts and neutrophil percentages were reduced following MSC therapy, accompanied by a relative recovery of lymphocyte proportions, suggesting attenuation of systemic inflammation.

Platelet counts were also affected by chronic TAA administration, showing a reduction of approximately 30–35% compared to controls. MSC-treated animals exhibited a partial restoration of platelet counts, further supporting an overall improvement in hematological homeostasis following regenerative intervention.

### 3.3. Histological Evaluation of Thioacetamide-Induced Liver Fibrosis

**Figure 3:**
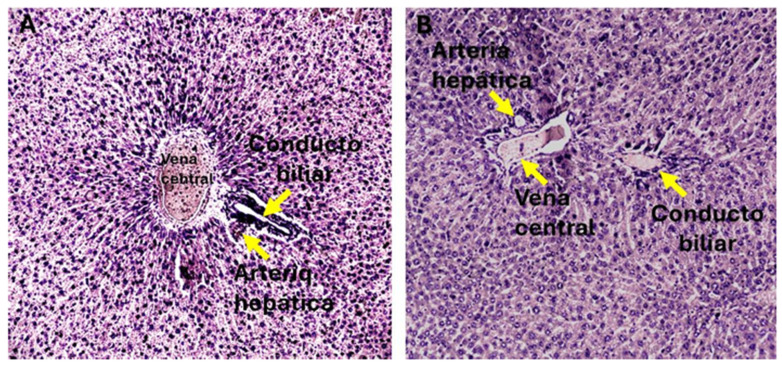
Histological evaluation of the liver in control group (G-I) animals. (**A**) Hematoxylin and eosin (H&E)–stained liver section showing preserved hepatic lobular architecture, with hepat cytes radially arranged around the central vein. Portal structures, including the hepatic artery an bile duct, are clearly identifiable (arrows). (**B**) Higher-magnification view highlighting the portal region with identifiable hepatic artery and bile duct within preserved hepatic parenchyma. Representative images were obtained using a 10× objective lens and a standard 10× ocular (total magnif cation, 100×). Representative fields were selected to illustrate normal hepatic architecture in the control group. The pathological evaluation was based on systematic analysis of hepatic archite ture using standardized morphological criteria across multiple sections and fields per animal, r ther than on a single representative image.

Chronic administration of thioacetamide (TAA) for 24 weeks induced histological changes consistent with liver fibrosis in animals from the G-II and G-III experimental groups. In contrast, control animals (G-I) treated with saline exhibited preserved hepatic architecture, characterized by normal lobular organization and well-defined portal structures, with no histological evidence of fibrosis (Figure 3).

**Figure 4:**
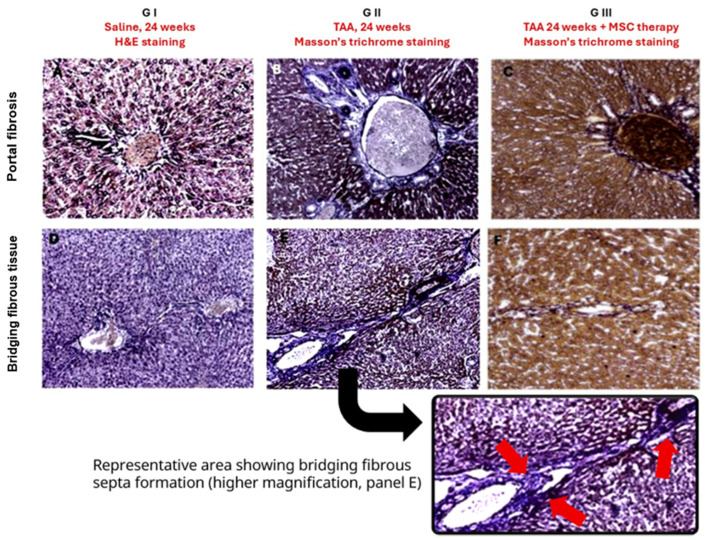
Histological and Histochemical Assessment of Liver Fibrosis Across Experimental Groups Representative liver sections from control (G-I), thioacetamide-induced fibrotic (G-II), and TAA + MSC-treated (G-III) animals are shown. Panels (**A**,**D**) (G-I) correspond to hematoxylin and eosin (H&E)-stained sections demonstrating preserved hepatic architecture in both portal (**A**) and centrolobular (**D**) regions, without septal formation. Panels (**B**,**E**) (G-II) correspond to Masson’s trichrome-stained sections from TAA-induced fibrotic animals. These panels show interlobular connective tissue expansion and bridging fibrous septa formation, characterized by collagen-rich septal structures extending between portal areas. Panels (**C**,**F**) (G-III) correspond to Masson’s trichrome-stained sections from TAA + MSC-treated animals, demonstrating attenuation of septal extension and partial architectural preservation compared to untreated fibrotic animals. Upper panels (**A**–**C**) represent portal regions, whereas lower panels (**D**–**F**) correspond to centrolobular regions. A higher-magnification inset derived from panel (**E**) is provided to clearly illustrate representative bridging fibrous septa connecting portal regions (red arrows), facilitating architectural interpretation. Histological evaluation was based primarily on fibrosis architecture and septal distribution rather than staining intensity. All micrographs were acquired at identical magnification using a 10× objective and a standard 10× ocular (total magnification, 100×).

Histochemical evaluation using Masson’s trichrome staining enabled the identification and classification of liver fibrosis severity according to the METAVIR scoring system adapted for rat liver tissue. As summarized in Table 3, all animals in the control group were classified as F0, confirming the absence of fibrosis. Representative H&E- and Masson’s trichrome–stained sections illustrating preserved architecture in control livers are shown in Figure 3 and Figure 4A,D.

In the G-II group, subjected to chronic TAA administration without subsequent treatment, Masson’s trichrome staining revealed marked collagen deposition predominantly in portal areas and fibrous septa (Figure 4B,E). Semi-quantitative assessment indicated mild to moderate portal fibrosis, with 57.1% of animals classified as F1 and 42.9% as F2, and no animals classified as F0 or advanced stages (F3–F4) (Table 3).

In contrast, animals in the G-III group, which received mesenchymal stem cell therapy following fibrosis induction, exhibited a redistribution of fibrosis stages. Masson’s trichrome staining demonstrated a visible reduction in collagen deposition and fibrotic septa (Figure 4C,F). Accordingly, 28.6% of animals were classified as F0 and 71.4% as F1, with no animals presenting moderate or advanced fibrosis (Table 3).

Overall, the histological and semi-quantitative findings demonstrate an attenuation of TAA-induced liver fibrosis in animals treated with mesenchymal stem cells compared with untreated fibrotic controls.

### 3.4. Histopathological Features Associated with Chronic Liver Injury

**Figure 5:**
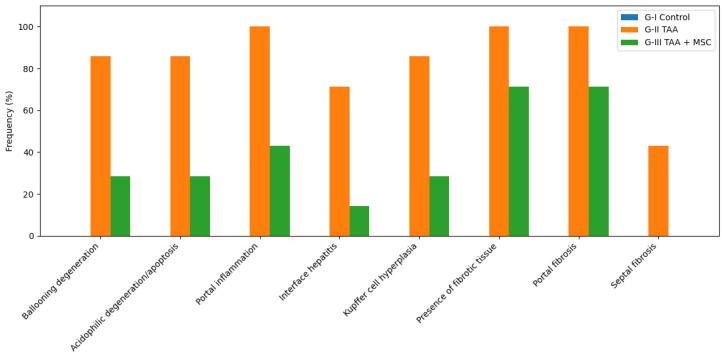
Frequency of histopathological liver alterations across experimental groups. The bar chart summarizes the percentage of animals in each experimental group exhibiting specific histopathological features of liver injury, inflammation, and fibrosis. Hepatocellular injury (ballooning degeneration and acidophilic degeneration/apoptosis), inflammatory changes (portal inflammation, interface hepatitis, and Kupffer cell hyperplasia), and fibrotic alterations (presence of fibrotic tissue, portal fibrosis, and septal fibrosis) were evaluated in liver sections stained with hematoxylin and eosin and Masson’s trichrome. Values represent the proportion of animals presenting each lesion within the Control (G-I), TAA-induced fibrosis (G-II), and TAA + MSC treatment (G-III) groups.

In addition to fibrosis staging, a detailed histopathological assessment was performed to characterize hepatocellular injury, inflammatory responses, and fibrotic changes across experimental groups. The frequency of histopathological findings is summarized in Table 4 and Figure 5.

Control animals (G-I) showed no evidence of hepatocellular degeneration, inflammatory infiltrates, or fibrotic changes. All evaluated histopathological features were absent in this group, consistent with preserved liver architecture observed in histological sections.

In contrast, animals subjected to chronic TAA administration (G-II) exhibited a high frequency of hepatocellular injury and inflammatory alterations. Ballooning degeneration and acidophilic degeneration/apoptosis were observed in 85.7% of animals, while portal inflammation was present in all animals (100%). Interface hepatitis and Kupffer cell hyperplasia were also frequently detected. Fibrotic changes were prominent in this group, with all animals exhibiting portal fibrosis and fibrotic tissue deposition, and 42.9% showing septal fibrosis.

Following mesenchymal stem cell therapy (G-III), a marked reduction in the frequency of histopathological alterations was observed. The prevalence of hepatocellular degeneration, inflammatory infiltrates, and Kupffer cell hyperplasia was substantially lower compared with untreated fibrotic animals. Although fibrotic tissue and portal fibrosis remained detectable in a proportion of animals, septal fibrosis was no longer observed in this group.

Prior to in vivo administration, the mesenchymal stromal cell (MSC) batch used in this study was immunophenotypically characterized by the certified supplier in accordance with the minimal criteria established by the International Society for Cellular Therapy (ISCT). Flow cytometry analysis confirmed ≥95% expression of CD73, CD90, and CD105 and ≤2% expression of CD45, CD34, CD19, and HLA-DR. Detailed quantitative data are provided in Supplementary Table S1, and representative flow cytometry histograms are shown in Supplementary Figure S1.

Overall, these findings indicate that mesenchymal stem cell therapy was associated with an attenuation of hepatocellular injury, inflammatory responses, and fibrotic alterations induced by chronic TAA exposure.

## 4. Discussion

The present study provides integrated experimental evidence demonstrating that mesenchymal stem cell (MSC) administration exerts a biologically relevant modulatory effect in a model of established chronic liver fibrosis induced by prolonged thioacetamide exposure. By combining biochemical, hematological, and histopathological analyses, the findings allow a comprehensive evaluation of both hepatic and systemic responses to MSC therapy under conditions of sustained fibrotic injury.

In line with previous characterizations of the thioacetamide model as a robust system for inducing progressive chronic liver injury, untreated animals in this study exhibited persistent hepatocellular damage and functional impairment. This was reflected by an approximately three- to four-fold increase in serum transaminase levels, a marked alteration of hepatic synthetic function—with albumin and total protein concentrations declining to below 70% of control values—and characteristic hematological disturbances, including moderate anemia and pronounced leukocytosis.

Histologically, the majority of untreated fibrotic animals exhibited early-to-intermediate METAVIR stages (F1–F2), characterized by portal fibrosis and early fibrous septa formation, without progression to cirrhotic stages. These findings confirm the establishment of chronic and sustained liver fibrosis of moderate severity, consistent with standardized descriptions of the thioacetamide model under comparable experimental conditions [1,17].

Following MSC administration, a consistent attenuation of liver injury was observed across all evaluated domains. At the biochemical level, transaminase levels were reduced by approximately 40–45% compared with untreated fibrotic animals, indicating partial restoration of hepatocellular integrity. However, enzyme values remained above baseline, which is consistent with current concepts of fibrosis reversibility emphasizing disease modulation rather than complete regression once chronic fibrotic remodeling is established [18,19]. Concordantly, hepatic synthetic function showed partial recovery, with albumin and total protein concentrations reaching approximately 85–90% of control values, suggesting functional stabilization rather than full normalization. Similar patterns of partial biochemical recovery have been reported in experimental antifibrotic interventions, supporting the notion that established fibrotic architecture may limit the extent of functional recovery [5,20].

Beyond liver-specific parameters, MSC therapy also influenced systemic manifestations associated with chronic liver injury. Thioacetamide-induced anemia, characterized by an approximate 20–25% reduction in erythroid parameters, and leukocytosis, with increases approaching 70–80% relative to controls, were partially attenuated following MSC administration. This trend toward hematological normalization suggests a systemic immunomodulatory effect that extends beyond the hepatic compartment. Such findings are consistent with the well-documented ability of MSCs to modulate cytokine networks, regulate immune cell activation, and attenuate chronic inflammation associated with fibrogenesis [21,22].

Histopathological evaluation provided further evidence supporting the antifibrotic modulatory effect of MSC therapy. Untreated fibrotic animals predominantly exhibited early-to-intermediate METAVIR stages (F1–F2), characterized by portal fibrosis and the presence of early fibrous septa, without progression to advanced or cirrhotic stages. In MSC-treated animals, histological analysis revealed a reduction in the extent and distribution of fibrotic tissue, accompanied by partial preservation of hepatic architecture. Importantly, residual fibrotic structures persisted despite therapy, indicating that MSC administration attenuates fibrogenic activity and limits fibrotic progression rather than inducing complete structural reversal. These observations are consistent with experimental evidence indicating that fibrosis at established early-to-intermediate stages may be modulated but not fully resolved under regenerative or antifibrotic interventions [18,19,23].

An important consideration in this study is the use of human umbilical cord–derived mesenchymal stromal cells in a rat model. It is well established that MSCs exhibit limited in vivo persistence and are rapidly cleared after administration, even in autologous or allogeneic settings (Galipeau and Sensébé, 2018) [24]. Accordingly, the therapeutic effects observed here are not attributed to long-term engraftment, differentiation, or tissue integration. Instead, they are consistent with a transient, paracrine-mediated mechanism of action, as originally conceptualized in the “injury drugstore” model [23], whereby MSCs exert short-lived immunomodulatory and antifibrotic effects through the release of bioactive mediators. This mode of action provides a biologically plausible explanation for the partial attenuation of fibrosis and functional improvement observed, even in an interspecies context.

Although tracking the biodistribution and persistence of administered mesenchymal stromal cells may provide valuable information in studies focused on cell engraftment or tissue replacement, this was not the primary objective of the present work. Given the widely accepted view that MSC-mediated effects are predominantly paracrine and transient, the therapeutic outcomes observed here were not interpreted as dependent on long-term cell survival or specific tissue localization. Future studies incorporating cell-labeling or tracking approaches may help further characterize early MSC–host interactions and biodistribution dynamics, particularly in the context of different routes of administration.

From a mechanistic standpoint, the therapeutic effects observed are most plausibly attributed to the paracrine and immunomodulatory properties of MSCs rather than direct replacement of damaged hepatocytes. MSC-derived soluble factors and extracellular vesicles have been shown to suppress hepatic stellate cell activation, modulate key inflammatory pathways, and promote endogenous repair processes, thereby creating a microenvironment conducive to fibrosis attenuation and functional improvement [6,23]. The coherence between biochemical, hematological, and histological findings observed in the present study supports this mechanistic framework and reinforces the role of MSCs as modulators of disease activity rather than drivers of complete tissue regeneration.

One limitation of this study is the relatively small sample size, which may reduce the statistical power to detect subtle effects. Therefore, statistical analyses were conducted within an exploratory framework, and the results should be interpreted primarily in terms of their biological relevance and pathophysiological coherence rather than as definitive inferential conclusions. The consistency observed across biochemical, hematological, and histological parameters supports the robustness of the biological trends identified in this preclinical model.

A limitation of this study is the lack of direct assessment of MSC biodistribution and persistence within liver tissue. Nevertheless, accumulating evidence indicates that MSC-mediated therapeutic effects in liver disease are largely driven by paracrine and immunomodulatory mechanisms rather than long-term engraftment. Previous studies have shown that systemically administered MSCs are short-lived and predominantly retained within the pulmonary circulation while maintaining significant biological activity through the secretion of soluble factors and extracellular vesicles [23,24,25]. These observations are consistent with the findings of the present study.

Liver fibrosis assessment in this study was based on an integrated histological approach, combining Masson’s trichrome staining for collagen visualization with METAVIR semiquantitative scoring. This strategy provides a robust and reproducible evaluation of fibrosis severity and architectural distribution. Complementary techniques, such as Sirius Red staining—particularly under polarized light—could be incorporated in future studies to enable more detailed quantitative characterization of fibrillar collagen and deeper mechanistic insights.

The present study focused on an integrated functional and histopathological evaluation of liver injury and its modulation by MSC therapy. While molecular, cellular, and proteomic analyses—such as α-SMA, type I collagen, and profibrotic cytokines—could provide additional mechanistic insights, these analyses were beyond the scope of the present work and represent a relevant direction for future investigations aimed at elucidating the underlying antifibrotic mechanisms.

Although the underlying mechanisms were not directly assessed in the present study, the histological and biochemical improvements observed following MSC therapy can be interpreted in light of previously described modulatory effects on the hepatic inflammatory microenvironment, inhibition of hepatic stellate cell activation, and regulation of key profibrotic pathways such as TGF-β signaling. In addition, MSCs have been shown to exert antifibrotic effects predominantly through paracrine and immunomodulatory mechanisms. Future studies will focus on directly evaluating these pathways to further elucidate the mechanisms underlying the observed therapeutic effects.

Taken together, the findings of this study place MSC therapy within a realistic biological and translational context. Rather than inducing complete reversal of established chronic liver fibrosis, MSC administration promotes partial functional recovery, reduces fibrotic burden, and attenuates systemic inflammation. These effects are consistent with the current consensus that liver fibrosis is a dynamic but incompletely reversible process, particularly once chronic fibrotic remodeling is established [1,26]. Accordingly, MSC-based interventions may hold their greatest therapeutic value as adjunctive strategies aimed at modulating disease activity, slowing fibrotic progression, and stabilizing liver function when integrated with etiological treatments or antifibrotic agents.

The present study demonstrates significant histopathological improvement following MSC administration in an experimental model of chronic liver fibrosis. Although specific cell-tracking strategies were not incorporated to assess long-term tissue persistence, the experimental design was primarily focused on evaluating global therapeutic effects on hepatic architecture and biochemical parameters. Accumulating experimental and preclinical evidence indicates that the antifibrotic activity of MSCs is predominantly mediated through paracrine and immunomodulatory mechanisms rather than permanent engraftment within the hepatic parenchyma. Multiple studies have shown that even when cellular persistence is transient, significant therapeutic effects occur through modulation of hepatic stellate cell activation, inhibition of profibrotic pathways such as TGF-β, attenuation of inflammatory signaling, and secretion of extracellular vesicles and trophic factors. In this context, the reduction of septal fibrosis and inflammatory alterations observed in the MSC-treated group is biologically consistent with this well-established mechanistic framework. Future studies incorporating cell-tracking approaches would further clarify MSC persistence dynamics in this model.

## 5. Conclusions

In conclusion, the present study demonstrates that mesenchymal stem cell (MSC) administration exerts a biologically relevant modulatory effect in a model of established chronic liver fibrosis induced by prolonged thioacetamide exposure. MSC therapy resulted in consistent improvements across biochemical, hematological, and histopathological parameters, reflecting partial recovery of liver function, attenuation of systemic inflammation, and reduction in the extent of fibrotic remodeling.

Although MSC administration did not induce complete reversal of established fibrosis, the observed reductions in serum transaminases, partial restoration of hepatic synthetic function, and attenuation of hematological alterations indicate meaningful disease modulation. Histopathological findings further support a reduction in fibrotic burden and limitation of fibrogenic progression within early-to-intermediate METAVIR stages (F1–F2), consistent with a modulatory rather than curative therapeutic effect.

Collectively, these results position MSC-based therapy as a promising adjunctive approach for modulating disease activity and stabilizing liver function in chronic liver fibrosis, rather than as a definitive curative intervention. By integrating functional, systemic, and structural outcomes, this study provides robust experimental evidence supporting the potential role of MSCs in cell-based strategies aimed at attenuating fibrogenic progression. Further studies addressing optimized dosing regimens, mechanistic pathways, and long-term outcomes will be essential to fully define the translational potential of MSC-based therapies in chronic liver disease.

## Figures and Tables

**Table 1:** Serum biochemical parameters by experimental group.

Parameter	Control (Saline, Week 25)	TAA Fibrosis (Week 25)	TAA + MSC (Week 32)	*p*-Value
AST (U/L)	82 ± 15	245 ± 40	138 ± 28	*p* = 0.004
ALT (U/L)	48 ± 10	198 ± 35	112 ± 22	*p* = 0.0008
ALP (U/L)	120 ± 18	310 ± 55	185 ± 32	*p* = 0.011
GGT (U/L)	9 ± 3	42 ± 8	22 ± 5	*p* = 0.006
Albumin (g/dL)	4.1 ± 0.3	2.8 ± 0.4	3.6 ± 0.3	*p* = 0.048
Total proteins (g/dL)	6.8 ± 0.4	5.4 ± 0.5	6.2 ± 0.4	*p* = 0.091
Glucose (mg/dL)	105 ± 12	88 ± 10	98 ± 11	*p* = 0.137

Note: Values represent the mean of individual measurements obtained from animals within each experimental group and are expressed as mean ± SD. Statistical significance was assessed using one-way analysis of variance (ANOVA). Reported *p*-values correspond to overall comparisons among experimental groups. AST, aspartate aminotransferase; ALT, alanine aminotransferase; ALP, alkaline phosphatase; GGT, gamma-glutamyl transferase; TAA, thioacetamide; MSC, mesenchymal stem cells.

**Table 2:** Hematological parameters by experimental group.

Parameter	Control (Saline, Week 25)	TAA Fibrosis (Week 25)	TAA + MSC (Week 32)	*p*-Value
Red blood cells (×10^6^/µL)	7.8 ± 0.5	6.1 ± 0.6	7.0 ± 0.5	*p* = 0.031
Hemoglobin (g/dL)	14.8 ± 0.9	11.6 ± 1.1	13.4 ± 0.8	*p* = 0.007
Hematocrit (%)	44.5 ± 3.2	35.8 ± 3.5	41.2 ± 3.0	*p* = 0.018
White blood cells (×10^3^/µL)	8.2 ± 1.1	14.6 ± 2.3	10.1 ± 1.5	*p* < 0.001
Neutrophils (%)	28 ± 5	46 ± 7	34 ± 6	*p* < 0.001
Lymphocytes (%)	64 ± 6	44 ± 8	56 ± 7	*p* = 0.014
Platelets (×10^3^/µL)	780 ± 95	520 ± 110	680 ± 100	*p* = 0.052

Note: Values represent the mean of individual measurements obtained from animals within each experimental group and are expressed as mean ± SD. Statistical significance was assessed using one-way analysis of variance (ANOVA). Reported *p*-values correspond to overall comparisons among experimental groups. TAA, thioacetamide; MSC, mesenchymal stem cells.

**Table 3:** Percentage distribution of liver fibrosis stages according to the METAVIR scoring system.

Experimental Group	F0 (%)	F1 (%)	F2 (%)	F3–F4 (%)
G-I Control (Saline)	100.0	0.0	0.0	0.0
G-II TAA-induced fibrosis	0.0	57.1	42.9	0.0
G-III TAA + MSC therapy	28.6	71.4	0.0	0.0

Note: Liver fibrosis was assessed semi-quantitatively using the METAVIR scoring system adapted for rat liver tissue on Masson’s trichrome–stained sections. Values represent the percentage of animals classified at each fibrosis stage within the corresponding experimental group.

**Table 4:** Frequency of histopathological liver findings by experimental group.

Histopathological Feature	G-I Control (%)	G-II TAA (%)	G-III TAA + MSC (%)
Hepatocellular injury			
Ballooning degeneration	0.0	85.7	28.6
Acidophilic degeneration/apoptosis	0.0	85.7	28.6
Inflammation			
Portal inflammation	0.0	100.0	42.9
Interface hepatitis (periportal)	0.0	71.4	14.3
Kupffer cell hyperplasia	0.0	85.7	28.6
Fibrotic changes			
Presence of fibrotic tissue	0.0	100.0	71.4
Portal fibrosis	0.0	100.0	71.4
Septal fibrosis (fibrous bridges)	0.0	42.9	0.0

Note: Values represent the percentage of animals within each experimental group exhibiting the indicated histopathological feature. Assessments were performed on liver sections stained with hematoxylin and eosin and Masson’s trichrome. A feature was considered present when clearly identified in at least one representative section per animal.

## Data Availability

The original contributions presented in this study are included in the article/Supplementary Materials. Further inquiries can be directed to the corresponding author(s).

## Supplementary Materials

The following supporting information can be downloaded at: https://www.mdpi.com/article/10.3390/diseases14030108/s1, Figure S1: title Representative flow cytometry histograms showing immunophenotypic characterization of human MSCs; Table S1: Individual serum biochemical parameters per animal; Table S2: Individual histopathological METAVIR fibrosis scores per animal; Table S3: Immunophenotypic characterization of MSCs by flow cytometry.
